# Resting and active motor thresholds versus stimulus–response curves to determine transcranial magnetic stimulation intensity in quadriceps femoris

**DOI:** 10.1186/1743-0003-11-40

**Published:** 2014-03-21

**Authors:** John Temesi, Mathieu Gruet, Thomas Rupp, Samuel Verges, Guillaume Y Millet

**Affiliations:** 1 Laboratoire de Physiologie de l’Exercice, Université de Lyon, Saint-Etienne F-42023, France; 2 Laboratoire HP2, Université Joseph Fourier & CHU Grenoble, Grenoble F-38000, France; 3 U1042, INSERM, Grenoble F-38000, France; 4 Human Performance Laboratory, Faculty of Kinesiology, University of Calgary, University Drive NW, Calgary, AB, Canada; 5 Laboratoire Motricité Humaine, Education, Sport, Santé, Université de Toulon, Toulon F-83000, France

**Keywords:** Stimulus intensity determination, Fatigue, Methodological considerations

## Abstract

**Background:**

Transcranial magnetic stimulation (TMS) is a widely-used investigative technique in motor cortical evaluation. Recently, there has been a surge in TMS studies evaluating lower-limb fatigue. TMS intensity of 120-130% resting motor threshold (RMT) and 120% active motor threshold (AMT) and TMS intensity determined using stimulus–response curves during muscular contraction have been used in these studies. With the expansion of fatigue research in locomotion, the *quadriceps femoris* is increasingly of interest. It is important to select a stimulus intensity appropriate to evaluate the variables, including voluntary activation, being measured in this functionally important muscle group. This study assessed whether selected quadriceps TMS stimulus intensity determined by frequently employed methods is similar between methods and muscles.

**Methods:**

Stimulus intensity in *vastus lateralis*, *rectus femoris* and *vastus medialis* muscles was determined by RMT, AMT (i.e. during brief voluntary contractions at 10% maximal voluntary force, MVC) and maximal motor-evoked potential (MEP) amplitude from stimulus–response curves during brief voluntary contractions at 10, 20 and 50% MVC at different stimulus intensities.

**Results:**

Stimulus intensity determined from a 10% MVC stimulus–response curve and at 120 and 130% RMT was higher than stimulus intensity at 120% AMT (lowest) and from a 50% MVC stimulus–response curve (*p* < 0.05). Stimulus intensity from a 20% MVC stimulus–response curve was similar to 120% RMT and 50% MVC stimulus–response curve. Mean stimulus intensity for stimulus–response curves at 10, 20 and 50% MVC corresponded to approximately 135, 115 and 100% RMT and 180, 155 and 130% AMT, respectively. Selected stimulus intensity was similar between muscles for all methods (*p* > 0.05).

**Conclusions:**

Similar optimal stimulus intensity and maximal MEP amplitudes at 20 and 50% MVC and the minimal risk of residual fatigue at 20% MVC suggest that a 20% MVC stimulus–response curve is appropriate for determining TMS stimulus intensity in the *quadriceps femoris*. The higher selected stimulus intensities at 120-130% RMT have the potential to cause increased coactivation and discomfort and the lower stimulus intensity at 120% AMT may underestimate evoked responses. One muscle may also act as a surrogate in determining optimal *quadriceps femoris* stimulation intensity.

## Background

Transcranial magnetic stimulation (TMS) is a safe non-invasive technique employed to investigate motor cortical function. A rapidly changing magnetic field is produced by a coil placed over the target area of the brain and this causes electromagnetic induction to generate an electrical current in the brain. When sufficiently strong, this electrical current causes direct and trans-synaptic depolarization, and stimulation, of the pyramidal tract axons.

Selection of suitable TMS intensity is an important concern for researchers and clinicians. While being non-invasive, stimulation of the brain may be uncomfortable, particularly at high stimulus intensities. Thus, reducing the number of stimuli necessary to determine stimulus intensity and selecting the minimum intensity necessary to appropriately measure the desired parameters is beneficial to both investigators and subjects. The latter point has been largely absent in the literature despite several studies finding either similar or contradictory results when two different stimulus intensities were employed [1-3]. The majority of recent research has been conducted on clinical populations, and thus, recommendations are generally directed towards investigations in clinical populations or for clinical purposes [4,5]. International Federation of Clinical Neurophysiology (IFCN) practical guidelines [5] discuss different methods of determining cortical motor threshold in relaxed muscle (RMT, resting motor threshold) and subsequent implications for stimulus intensity. These practical guidelines state that optimal intensity for TMS should correspond to the transition from the rising slope to the flat portion of the sigmoid stimulus–response (stimulator intensity-elicited motor-evoked potential (MEP) amplitude) curve and that this optimal intensity corresponds approximately to 140% RMT or 170% cortical motor threshold determined during voluntary muscular contraction (AMT, active motor threshold) [5]. Stimulus–response curves are not routinely used for diagnostic purposes despite providing a direct means to determine stimulus intensity to elicit maximal MEP responses. This type of method has recently been employed by several research groups in the applied exercise sciences [2,3,6-8] while several other studies have determined stimulus intensity from RMT or AMT [9-12]. It remains to be determined if commonly employed selection of TMS intensity as determined by RMT, AMT and stimulus–response curves in this applied field result in selection of similar TMS intensities. Furthermore, practical guidelines for TMS intensity determination are normally based on investigations in upper-limb muscles. Data from lower-limb muscles are limited despite the functional importance of the lower limbs, specifically in regards to locomotion.

Studies utilizing TMS to investigate fatigue or acute exercise interventions in lower-limb muscles have used various methods to determine stimulus intensity. The most common of these has been RMT (the lowest intensity necessary to elicit MEPs, usually of at least 0.05 mV in amplitude, in at least one half of a given number of stimuli in the relaxed muscle) [9,11,13-15]. Another common method is AMT (the lowest intensity necessary to elicit detectable MEPs or MEPs of a pre-determined amplitude in at least one half of a given number of stimuli during weak voluntary contraction) [10,12,16-18]. More recently, numerous studies have selected a stimulus intensity to evoke MEP responses of a certain size in the target muscle during voluntary contraction [2,6-8,19,20]. Some studies are unclear about the intensity chosen for TMS [21] or whether intensity determination was performed with the muscle in the relaxed or contracted state [22]. Other studies based stimulus intensity on the intensity chosen to stimulate another muscle group [23] or simply selected maximal stimulator output [24].

Each of these methods produces a unique set of concerns. Cortical excitability is intrinsically linked to voluntary contraction intensity. While cortical excitability is low at rest, it increases rapidly as contraction intensity increases from rest [25,26]. Whether determination of stimulus intensity in relaxed muscle (as with RMT) is appropriate for conducting measures in contracting muscle is unknown. Similarly, it remains to be determined whether selecting stimulus intensity at a different contraction level than that employed during evaluation is appropriate.

An additional complexity when evaluating leg muscles (e.g. knee extensors, knee flexors, plantar flexors) is that, unlike the elbow flexors, there is not a single dominant muscle. Whether it is appropriate to use a single muscle as a surrogate for all muscles within a muscle group (e.g. *rectus femoris* [RF] for the *quadriceps femoris*) when determining stimulus intensity remains to be investigated, especially since muscles and muscle groups may respond differently to TMS. This is a pertinent issue given both the functional importance of the *quadriceps femoris* and its increasing prevalence in studies utilizing TMS in the evaluation of fatigue [2,7-9,27].

Fatigue of the quadriceps is increasingly being evaluated in both healthy and clinical populations. An important measure in fatigue evaluation is voluntary activation (VA) [28,29]. Evaluation of cortical VA utilizes superimposed twitches (SIT) evoked by TMS delivered during moderate- to high-intensity voluntary contractions (i.e. ≥50% maximal voluntary force [MVC]) [20,30,31]. Evoked MEP responses at ~50% MVC are theoretically maximal due to the firing of almost all motoneurons and maximal corticospinal excitability [20,25,31]. Since a key component of VA is the requirement that the muscle is driven maximally, maximal MEP amplitude is believed to be essential to ensure that SIT, and by extension VA, is not underestimated. Recently, *quadriceps femoris* studies have begun using TMS-induced antagonist coactivation as a criterion in the selection of TMS intensity [6-9] since this may cause SIT underestimation, and thus underestimate the development of central fatigue.

A comparison of selected stimulus intensity between published studies is impossible due to the use of different methods and equipment and different study aims. Thus, the primary objective of this study was to compare different methods of determining TMS intensity for the purposes of fatigue evaluation in the *quadriceps femoris* on selected stimulus intensity. Because of the use of voluntary contractions ≥50% MVC to determine VA and because maximal MEP responses have been observed to occur during contractions of approximately 50% MVC, a stimulus–response curve at 50% MVC was used as a baseline for comparison with other methods (i.e. this method most closely resembles fatigue evaluation). By using the same stimulator, coil and stimulation site, this protocol permits the isolation of differences between methods of stimulus intensity determination. The secondary objective was to determine whether selected stimulus intensity is similar for each of the three superficial quadriceps muscles.

## Methods

### Subjects

Eight healthy active men participated in this study (means ± standard deviation: age, 30 ± 8 years; height, 181 ± 5 cm; body mass, 73 ± 4 kg). Subjects were informed of the experimental protocol and all associated risks prior to giving written informed consent as part of a medical inclusion. All procedures conformed to the Declaration of Helsinki and were approved by the Comité de Protection des Personnes Sud-Est 1, France.

### Experimental design

Each subject completed one familiarization session and one experimental session. During the familiarization session, subjects were introduced to all procedures conducted in the experimental session and repeated trials until they performed all tests consistently and as directed. The largest MVC from the familiarization session was used to calculate contraction intensities and the reproducibility of MVC between sessions was verified.

### Force and electromyographic recordings

Knee extensor force was measured during voluntary and evoked contractions by a calibrated force transducer (Meiri F2732 200 daN, Celians, Montauban, France) with amplifier that was attached by a non-compliant strap to the right leg immediately proximal to the malleoli of the ankle joint. Subjects were seated upright in a custom-built chair with both hips and right knee at 90° of flexion. The force transducer was fixed to the chair such that force was measured in direct line to the applied force. Electromyographic (EMG) activity of the right knee extensors (RF, *vastus lateralis* [VL] and *vastus medialis* [VM]) and flexors (*biceps femoris*, [BF]) was recorded.

EMG activity was recorded with a pair of self-adhesive surface (10-mm recording diameter) electrodes (Meditrace 100, Covidien, Mansfield, USA) in bipolar configuration with a 30-mm interelectrode distance and the reference on the patella. Low impedance (<5 kΩ) between electrodes was obtained by shaving, gently abrading the skin with sandpaper and then cleaning it with isopropyl alcohol. Signals were analogue-to-digitally converted at a sampling rate of 2000 Hz by PowerLab system (16/30—ML880/P, ADInstruments, Bella Vista, Australia) and octal bio-amplifier (ML138, ADInstruments) with bandpass filter (5–500 Hz) and analyzed offline using Labchart 7 software (ADInstruments).

### Femoral nerve stimulation

Single electrical stimuli of 1-ms duration were delivered via constant-current stimulator (DS7A, Digitimer, Welwyn Garden City, Hertfordshire, UK) to the right femoral nerve via a 30-mm diameter surface cathode in the femoral triangle (Meditrace 100, Covidien, Mansfield, USA) and 50 x 90 mm rectangular anode (Durastick Plus, DJO Global, Vista, USA) on the *gluteus maximus*. Single stimuli were delivered incrementally until plateaus in maximal M-wave (Mmax) and twitch amplitude were reached. Three supramaximal stimuli at 130% of the intensity to produce maximal Mmax and twitch responses (52 ± 9 mA) were delivered at rest.

### Transcranial magnetic stimulation

Single-pulses (0.1-ms rise time; 1-ms duration) were manually delivered by TMS to elicit MEPs and twitches in the right knee extensors. The contralateral motor cortex was stimulated by a magnetic stimulator (Magstim 200^2^, The Magstim Company Ltd, Whitland, UK) with 110-mm double-cone coil (maximum output of 1.4 T) to induce a postero-anterior current. The coil was manually controlled by an experienced investigator throughout the protocol. Subjects wore a cervical collar during all TMS measures to stabilize the head and neck.

### Determination of coil position

Subjects wore a latex swim cap on which lines were drawn between the preauricular points and from nasion to inion to identify the vertex. Every centimeter from 1 cm anterior to 3 cm posterior to the vertex was demarcated along the nasal-inion line and also to 2 cm over the left motor cortex. At each point a stimulus was delivered at 70% maximal stimulator output during brief voluntary contraction of the knee extensors at 10% MVC. Target force was displayed on a screen and subjects were provided with real-time visual feedback during all voluntary contractions throughout the protocol. The coil was positioned at the site evoking the largest VL (39.5 ± 19.2% Mmax), RF (75.9 ± 26.7% Mmax) and VM (45.0 ± 21.3% Mmax) MEP amplitudes and SIT with minimal BF MEP amplitude. This coil position was drawn directly onto the swim cap and used throughout the protocol. Coil position was also verified before the delivery of each stimulus.

### Determination of stimulus intensity

Four methods of determining stimulus intensity were investigated in the following order: 1) RMT: Beginning at 30% of maximal stimulator output and increasing by 5% increments to 80%, subjects received 10 stimuli at each stimulus intensity with the knee extensors completely relaxed. Stimuli were delivered at 10-s intervals. 2) AMT/stimulus–response curve at 10% MVC: Subjects performed brief voluntary contractions (~2-3 s) of the knee extensors with TMS delivered 10 times at 20, 25, 30, 35 and then 40% of maximal stimulator output. Subjects then performed brief contractions with TMS delivered 4 consecutive times at each of the following randomly-ordered stimulus intensities: 50, 60, 70 and 80% of maximal stimulator output. All stimuli were delivered at 15-s intervals. 3) Stimulus–response curve at 20% MVC: Subjects performed brief contractions (~2-3 s) of the knee extensors with TMS delivered 4 consecutive times at each of the following randomly-ordered stimulus intensities: 20, 30, 40, 50, 60, 70 and 80% maximal stimulator output. Stimuli were delivered at 15-s intervals. 4) Stimulus–response curve at 50% MVC: Similar to the stimulus–response curve at 20% MVC except that stimuli were delivered at 20-s intervals. During voluntary contractions, TMS was always delivered once the subject had contracted to the appropriate force level and the force had stabilized [32] and 10 min rest was provided between each of the four methods.

### Data analysis

**Figure 1:**
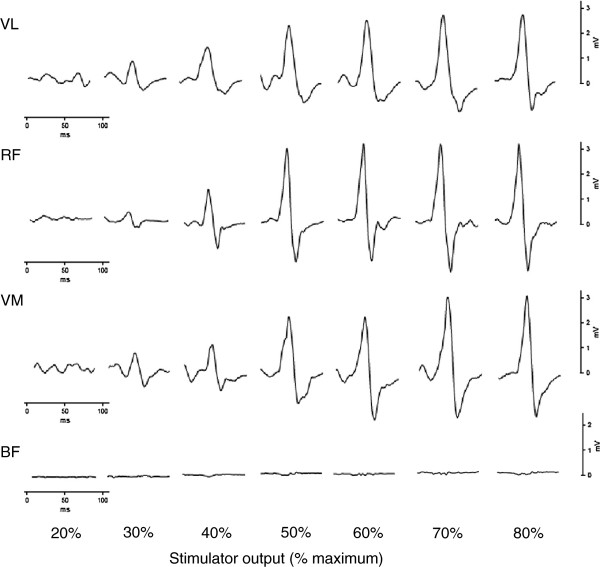
**Representative individual motor-evoked potentials from a stimulus–response curve.** Representative individual motor-evoked potentials elicited in the *vastus lateralis* (VL), *rectus femoris* (RF), *vastus medialis* (VM) and *biceps femoris* (BF) for one subject from a stimulus–response curve at 20% maximal voluntary force.

Peak-to-peak MEP and Mmax amplitudes were measured offline for each individual response. Individual MEP and Mmax amplitudes were then averaged and MEP amplitudes were normalized to Mmax amplitudes evoked in relaxed muscle. Data collected from a similar group of subjects in our laboratory indicated Mmax amplitudes were similar at rest and at the contraction intensities employed in this study (i.e. up to 50% MVC, unpublished observations, 2012). RMT was determined as the lowest stimulus intensity producing at least 5 MEPs of at least 0.05 mV from 10 stimuli. RMT was also determined from 6 and 8 stimuli (minimum of 3 and 4 MEPs, respectively). Stimulus intensities of 120 and 130% RMT were determined for comparison with methods used in other lower-limb studies [9,11,13,14,27,30,33]. AMT was determined by visual identification of MEPs above background EMG from contractions at 10% MVC [34] and corresponded to the lowest stimulus intensity producing MEPs in at least half the contractions. Classically, fixed thresholds are used to determine the presence of a MEP (i.e. 0.2 mV at 10% MVC [12]); however, the large variability in background EMG activity for the three measured quadriceps muscles rendered this method impractical. AMT was also determined from 6 and 8 stimuli (minimum of 3 and 4 MEPs, respectively). The stimulus intensity of 120% AMT was determined for comparison because of its use in other lower-limb studies [10,12]. Stimulus–response curves at 10, 20 and 50% MVC were used to determine stimulus intensity by identifying the minimum stimulus intensity to evoke maximal MEP amplitude (i.e. the lowest intensity resulting in an increase of less than 5% MEP amplitude at higher stimulus intensities). Individual MEPs from a typical stimulus–response curve at 20% MVC for one subject are presented in Figure 1. Antagonist MEP amplitude was examined to verify that this stimulus intensity did not elicit increased TMS-induced coactivation. For the 10% MVC stimulus–response curve, only the first 4 stimuli at 20, 30 and 40% maximal stimulator output were considered. Where a plateau was not reached, MEP amplitude at 80% maximal stimulator output was compared to the estimated maximal MEP amplitude from Boltzmann modeling (see next paragraph). If mean MEP amplitude was greater or equal to the maximal modeled MEP amplitude, 80% was accepted as being part of the plateau and selected as the appropriate stimulus intensity. Otherwise, a plateau was determined to not have occurred and the data was excluded from analyses.

MEP amplitude from stimulus–response curves were modeled with a Boltzmann sigmoidal function [35] using the equation:

$$\text{MEPmax}\left(S\right)=\frac{\text{MEPmax}}{1+\text{exp}\left[\frac{S50-S}{k}\right]}$$

where MEPmax is the estimated maximal MEP amplitude, *S* is the stimulus intensity, *S*50 is the stimulus intensity required to produce a response equal to half MEPmax and *k* is the slope parameter (inversely proportional to maximal function steepness). To eliminate the effects of background EMG in the modeling process, an amplitude of 0 mV was assigned to all responses in which there was no discernible MEP.

### Statistics

**Figure 2:**
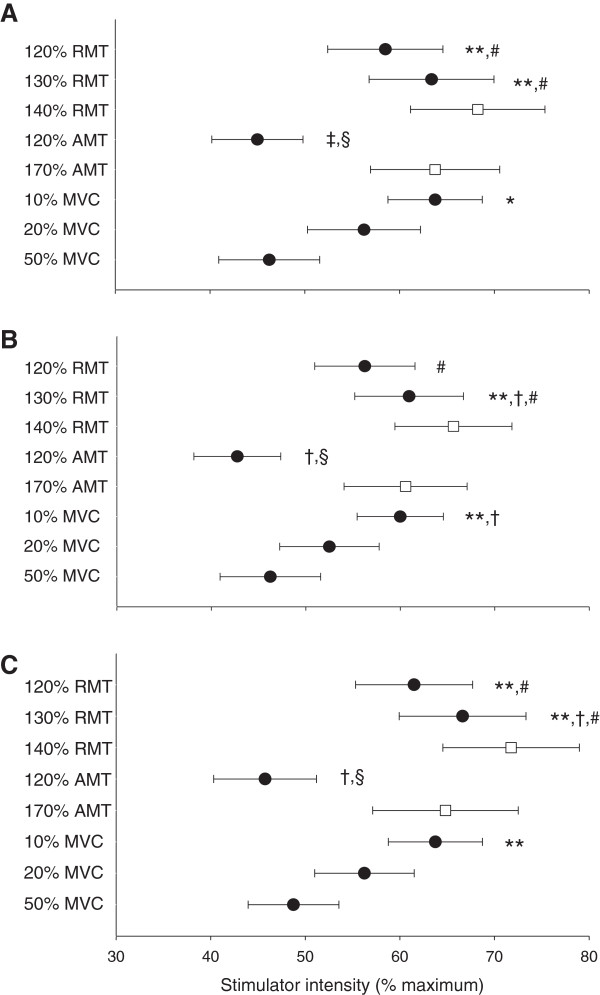
**Comparison of methods for determination of TMS stimulus intensity.** Comparison of different methods of determining TMS stimulus intensity for *vastus lateralis* in **Panel A**, *rectus femoris* (n = 7) in **Panel B** and *vastus medialis* in **Panel C**. The methods compared are resting motor threshold (RMT), active motor threshold (AMT) during contractions at 10% maximal voluntary force (MVC) and stimulus–response curves at 10, 20 and 50% MVC. Stimulus intensity is presented as means ± standard error of the mean for stimulus–response curves and commonly utilized intensities derived from thresholds (●) and estimated optimal intensity (□) [5]. Significantly different from 50% MVC, * (*p* < 0.05) and ** (*p* < 0.01); significantly different from 20% MVC, † (*p* < 0.05) and ‡ (*p* < 0.01); significantly different from 10% MVC, § (*p* < 0.01); significantly different from 120% AMT, # (*p* < 0.01).

Statistical analyses were performed with Statistica (version 8, Tulsa, USA). The Shapiro-Wilk test was used to verify data normality. One-way repeated measures analyses of variance (ANOVA) were used to evaluate the method of stimulus determination (120 and 130% RMT, 120% AMT and stimulus–response curves), any difference between muscles and the effect of contraction intensity on Boltzmann parameters. One-way repeated measures ANOVA were also used to compare AMT and RMT determined from 6, 8 and 10 stimuli. When ANOVA revealed significant interactions, the Newman-Keuls *post-hoc* test was used to identify differences. The effect size as determined from the ANOVA were calculated as ω^2^ to reduce potential bias associated with the small sample size [36]. Dependent t-tests were used to compare Boltzmann and linear relationships for the coefficient of determination of MEP amplitude. Statistical significance was set at *p* < 0.05. All data are expressed as means ± standard deviation except Figure 2 where values are expressed as means ± standard error of the mean.

## Results

### Selected stimulus intensity

One subject did not reach a plateau in MEP amplitude in RF with the 10% MVC stimulus–response curve and was thus excluded from all relevant analyses.

**Figure 3:**
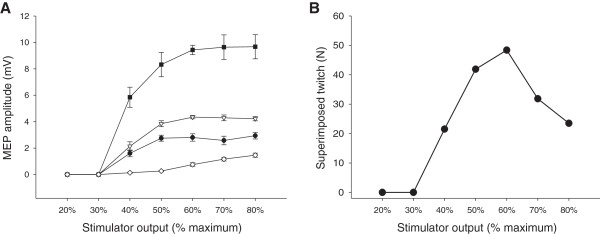
**Sample stimulus–response curves.** Stimulus–response curves at 50% maximal voluntary force for one subject for *vastus lateralis* (●), *rectus femoris* (∇)*, vastus medialis* (■) and *biceps femoris* (◊) in **Panel A** and superimposed twitch in **Panel B**. All values are presented as means ± standard deviation **(Panel A)** or means **(Panel B)** of four evoked responses at each stimulus intensity.

Neither AMT nor RMT were different whether determination occurred with the first 6, 8 or 10 responses at each stimulus intensity for any muscle (*p* > 0.05). Therefore all subsequent analyses were conducted based upon AMT and RMT determined from 10 stimuli at each stimulus intensity. Selected TMS intensity determined by RMT, AMT and stimulus–response curves are presented in Figure 2. Stimulus intensities determined from RMT (120 and 130%) and stimulus–response curves at 10% MVC were higher than the intensity determined by stimulus–response curve at 50% MVC (VL: F(5,35) = 8.54, *p* < 0.001, ω^2^ = 0.48; RF: F(5,30) = 8.13, *p* < 0.001, ω^2^ = 0.50; VM: F(5,35) = 7.69, *p* < 0.001, ω^2^ = 0.45). Stimulus intensity at 120% AMT was lower than stimulus intensity determined from stimulus–response curves at both 10 and 20% MVC (*p* < 0.05). Table 1 presents the selected stimulus intensities from the stimulus–response curves as a percentage of the stimulus intensity to elicit both RMT and AMT to contextualize the differences between these methods. There was also no difference in selected intensity between muscles for any method (RMT: *F*(2,14) = 2.62, *p* = 0.11, ω^2^ = 0.16; AMT: *F*(2,14) = 1.21, *p* = 0.33, ω^2^ = 0.02; 10% MVC: *F*(2,12) = 1.00, *p* = 0.40, ω^2^ = 0.00; 20% MVC: *F*(2,14) = 1.15, *p* = 0.35, ω^2^ = 0.02; 50% MVC: *F*(2,14) = 0.778, *p* = 0.48, ω^2^ = 0) nor difference in normalized MEP amplitude at the selected stimulus intensity between 10, 20 and 50% MVC stimulus–response curves (VL: *F*(2,14) = 3.23, *p* = 0.07, ω^2^ = 0.21; RF: *F*(2,12) = 2.48, *p* = 0.13, ω^2^ = 0.16; VM: *F*(2,14) = 2.81, *p* = 0.09, ω^2^ = 0.18) (Table 2,). Raw BF MEP amplitudes at the selected stimulus intensities were 0.51 ± 0.54, 0.53 ± 0.41 and 0.53 ± 0.41 mV for VL, 0.42 ± 0.47, 0.43 ± 0.41 and 0.54 ± 0.31 mV for RF and 0.40 ± 0.45, 0.45 ± 0.40 and 0.59 ± 0.42 mV for VM for 10, 20 and 50% MVC stimulus response curves, respectively. At the stimulus intensity selected by AMT determined during contractions at 10% MVC, raw BF MEP amplitudes were 0.30 ± 0.41, 0.28 ± 0.42 and 0.29 ± 0.42 mV for VL, RF and VM, respectively. A single stimulus–response curve at 50% MVC is presented in Figure 3.

**Table 1:** Selected stimulus intensity from stimulus–response curves presented as a percentage of stimulus intensity to elicit active and resting motor thresholds

		***Vastus lateralis***	***Rectus femoris***	***Vastus medialis***
10% MVC	RMT	135 ± 26	138 ± 26	129 ± 20
		(109 – 175)	(109 – 175)	(107 – 160)
	AMT	179 ± 48	187 ± 46	177 ± 46
		(120 – 250)	(120 – 250)	(117 – 250)
20% MVC	RMT	117 ± 27	113 ± 15	114 ± 16
		(86 – 175)	(86 – 133)	(92 – 140)
	AMT	154 ± 40	151 ± 32	156 ± 36
		(100 – 200)	(120 – 200)	(100 – 200)
50% MVC	RMT	96 ± 21	100 ± 23	98 ± 21
		(71 – 127)	(75 – 140)	(67 – 120)
	AMT	124 ± 22	131 ± 26	132 ± 27
		(100 – 150)	(100 – 175)	(86 – 160)

AMT: active motor threshold; MVC: maximal voluntary force; RMT: resting motor threshold. For *rectus femoris*, values from the stimulus–response curve at 10% MVC are n = 7. Values are expressed as means ± standard deviation and (range).

**Table 2:** Normalized motor-evoked potential amplitudes at selected stimulus intensity from stimulus–response curves for all quadriceps muscles

	***Vastus lateralis***	***Rectus femoris***	***Vastus medialis***
10% MVC	34.5 ± 20.5	75.8 ± 16.1	43.4 ± 21.6
	(13.2 – 75.9)	(53.3 – 98.7)	(14.3 – 82.1)
20% MVC	42.9 ± 16.7	82.8 ± 18.7	52.8 ± 18.9
	(22.9 – 69.2)	(58.6 – 111.0)	(16.9 – 82.6)
50% MVC	45.3 ± 11.1	85.9 ± 22.2	49.7 ± 10.8
	(28.1 – 63.3)	(62.4 – 130.5)	(30.2 – 69.5)

MVC: maximal voluntary force. For *rectus femoris*, values from the stimulus–response curve at 10% MVC are n = 7. Normalized motor-evoked potential amplitudes are expressed as means  ±  standard deviation and (range).

In VL, RF and VM, Mmax amplitudes were 16.2 ± 4.1 mV, 7.4 ± 1.8 mV and 17.0 ± 6.7 mV, respectively. Central drive as indicated by RMS · Mmax^-1^ for VL (0.0046 ± 0.0014), RF (0.0039 ± 0.0007) and VM (0.0053 ± 0.0025) at 10% MVC and VL (0.0088 ± 0.0024), RF (0.0086 ± 0.0019) and VM (0.0100 ± 0.0039) at 20% MVC were similar (*F*(2,14) = 1.32, *p* = 0.30, ω^2^ = 0.05, and *F*(2,14) = 0.660, *p* = 0.53, ω^2^ = 0, respectively). At 50% MVC, RMS · Mmax^-1^ for RF (0.0376 ± 0.0160) was greater than for both VL (0.0237 ± 0.0094) and VM (0.0264 ± 0.0115) (*F*(2,14) = 8.36, *p* = 0.004, ω^2^ = 0.00).

### Boltzmann sigmoidal curves

**Figure 4:**
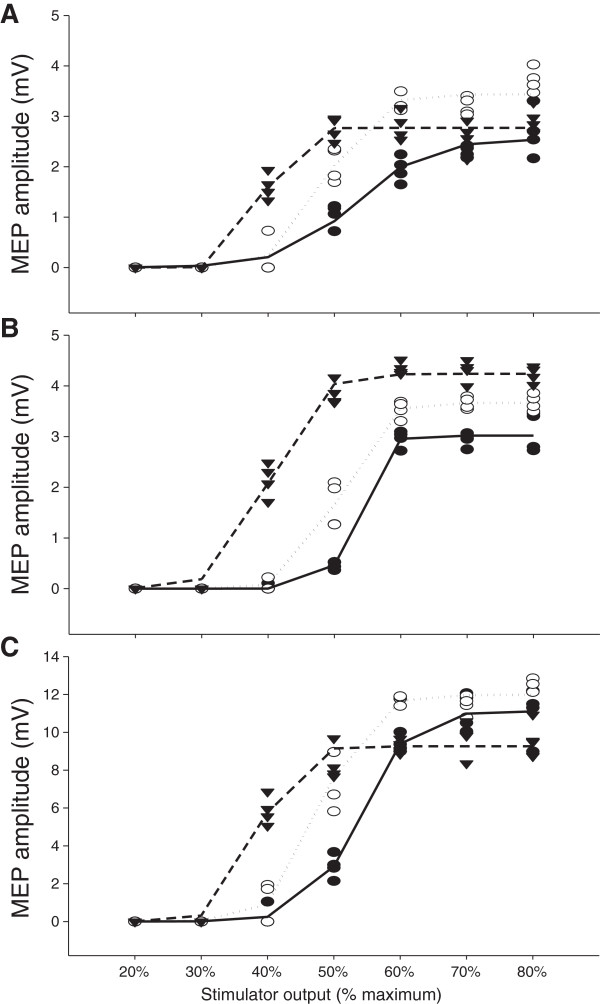
**Sample Boltzmann curves.** Boltzmann sigmoidal function plotted versus stimulator intensity for one subject for *vastus lateralis* in **Panel A**, *rectus femoris* in **Panel B** and *vastus medialis* in **Panel C**. All motor-evoked potentials used in the modeling and the Boltzmann curves are presented for stimulus–response curves at 10 (●,), 20 (○, ) and 50% (▼, ) of maximal voluntary force.

Boltzmann curves from a typical subject are presented in Figure 4. Boltzmann curves provided a significantly better fit for the relationship between MEP amplitude and stimulator intensity than a linear relationship for stimulus–response curves at 10, 20 and 50% MVC for all muscles (*p* < 0.05). As contraction intensity increased, *S*50 decreased in all muscles (VL: *F*(2,14) = 33.1, *p* < 0.001, ω^2^ = 0.79; RF: *F*(2,14) = 55.6, *p* < 0.001, ω^2^ = 0.87; VM: *F*(2,14) = 32.5, *p* < 0.001, ω^2^ = 0.79). Few differences were observed in MEPmax · Mmax^-1^ (only RF lower at 10% MVC; VL: *F*(2,14) = 1.88, *p* = 0.19, ω^2^ = 0.09; RF: *F*(2,14) = 3.88, *p* = 0.046, ω^2^ = 0.25; VM: *F*(2,14) = 2.40, *p* = 0.13, ω^2^ = 0.14) and k (only VL lower at 10% MVC; VL: *F*(2,14) = 7.50, *p* = 0.006, ω^2^ = 0.43; RF: *F*(2,14) = 1.62, *p* = 0.23, ω^2^ = 0.07; VM: *F*(2,14) = 0.911, *p* = 0.42, ω^2^ = 0). Results from modeling the stimulus–response curve data with the Boltzmann equation are presented in Table 3.

**Table 3:** **Modeled Boltzmann parameters for *vastus lateralis*, *rectus femoris *** **and *vastus medialis *** **muscles**

		***Vastus lateralis***	***Rectus femoris***	***Vastus medialis***
**MEPmax · Mmax**^**-1**^				
	10% MVC	34.7 ± 21.6	68.8 ± 19.6^*,#^	42.7 ± 22.3
	20% MVC	42.9 ± 17.1	83.3 ± 19.3	51.2 ± 18.7
	50% MVC	42.6 ± 11.1	83.0 ± 23.0	47.7 ± 11.9
***S*** **50**				
	10% MVC	43 ± 10^**,##^	45 ± 11^**,##^	44 ± 10^**,##^
	20% MVC	38 ± 11^**^	40 ± 10^**^	39 ± 10^**^
	50% MVC	32 ± 9	30 ± 7	34 ± 7
***k***				
	10% MVC	0.051 ± 0.018^**,#^	0.036 ± 0.029	0.037 ± 0.018
	20% MVC	0.032 ± 0.020	0.027 ± 0.015	0.031 ± 0.026
	50% MVC	0.020 ± 0.018	0.019 ± 0.014	0.027 ± 0.012
**r**^**2**^				
	10% MVC			
	Model	0.928 ± 0.045^†^	0.964 ± 0.051^‡^	0.937 ± 0.045^‡^
	Linear regression	0.804 ± 0.095	0.770 ± 0.118	0.779 ± 0.112
	20% MVC			
	Model	0.943 ± 0.048^†^	0.982 ± 0.012^‡^	0.933 ± 0.050^‡^
	Linear regression	0.724 ± 0.173	0.716 ± 0.180	0.688 ± 0.196
	50% MVC			
	Model	0.919 ± 0.052^‡^	0.900 ± 0.092^‡^	0.882 ± 0.115^‡^
	Linear regression	0.563 ± 0.214	0.537 ± 0.207	0.598 ± 0.190

MEPmax · Mmax^-1^: maximal motor-evoked potential amplitude (MEPmax) normalized to maximal M-wave amplitude, MVC: maximal voluntary force, *S*50: stimulus intensity to evoke motor-evoked potentials half the amplitude of MEPmax (as % maximal stimulator output), *k*: slope parameter (inversely proportional to maximal function steepness), r^2^: coefficient of determination. Values are expressed as means ± standard deviation. Significantly different from 50% maximal voluntary force (MVC), * (*p* < 0.05) and ** (*p* < 0.01); significantly different from 20% MVC, # (*p* < 0.05) and ## (*p* < 0.01); significantly different from linear relationship, † (*p* < 0.05) and ‡ (*p* < 0.01).

## Discussion

The main findings of this study are that (i) commonly-used stimulus intensities based upon RMT and a stimulus–response curve at 10% MVC are higher than those when determined using stimulus–response curves at 20 and 50% MVC and AMT and (ii) selected stimulus intensity, as determined by all methods, is similar between the three quadriceps muscles investigated. Because a stimulus–response curve performed at 20% MVC resulted in selection of a similar stimulus intensity to a stimulus–response curve at 50% MVC and because a stimulus–response curve at 20% MVC has a lower risk of inducing fatigue with repeated submaximal contractions, the present study indicates that this method is suitable for determining optimal stimulus intensity.

### Comparison of methods

#### ***Resting motor threshold***

In evaluation of the lower limbs to investigate fatigue or the effect of an exercise intervention, RMT has often been used to determine stimulus intensity. Most frequently this has been at 120 [11,13,14,33] and 130% RMT [9,27,30]. The present study found that the use of these RMT intensities results in selection of higher stimulus intensities than a stimulus–response curve at 50% MVC and that stimulus intensity at 130% RMT is significantly greater than that from a stimulus–response curve at 20% MVC. No studies in the lower limbs were found to employ the suggested IFCN equivalent of 140% RMT [5], an intensity higher than the intensity at the transition from the rising slope to the plateau of the stimulus–response curves in the present study (Table 1).

There are several concerns about using RMT to determine optimal stimulus intensity in fatigue studies. The most important is whether it is appropriate to determine stimulus intensity in the relaxed muscle when evaluation of TMS-related parameters is conducted during muscular contraction. The rapid increase in cortical excitability from rest to even very weak contraction [25,26] and the differential results in MEP evolution evaluated after fatiguing contractions when assessed in relaxed (i.e. decreased MEP amplitude/area [37-39]) and contracting (i.e. no change or increased MEP amplitude/area [10,40,41]) muscle present conceptual difficulties. More practically, increased stimulus intensity is associated with greater subject discomfort and this is important when recruiting healthy subjects and critical when evaluating clinical populations. If RMT is used to select stimulus intensity, no more than 6 stimuli should be delivered at each stimulus intensity since more stimuli do not better identify RMT contrary to the accepted standard of 10 stimuli at each intensity [5,42]. It has also been reported that extremely high stimulus intensities are often required to determine RMT due to low cortical excitability at rest and that in some subjects RMT cannot be determined [17]. This difficulty has also occurred in our laboratory. Given that high stimulus intensities may be required to evoke a MEP and the variable nature of MEP responses [43], particularly in the relaxed muscle [44], it may be difficult to identify an appropriate coil position.

Magnetic stimulation of the motor cortex with a double-cone coil permits more precise localization of specific brain areas than with a circular coil. It does not, however, permit localization with pin-point accuracy. Barker [45] detailed the induced electrical field and its rate of change with different coil types. Given that the motor cortex is not divided into discrete sections corresponding to individual muscles [46] and the imprecise area of stimulation with TMS, other muscle groups will inevitably be stimulated. Awiszus et al. [47] discussed the problem of high-intensity electrical muscle stimulation stimulating both agonist and antagonist muscles of the upper limb and these findings can likely be applied to transcranial motor cortical stimulation. To our knowledge, Todd et al. [31] were the first to specifically address this with a criterion in the determination of stimulus intensity (i.e. “a small MEP” in the antagonist). Figure 3 illustrates the 50% MVC stimulus–response curve of one subject. A plateau in quadriceps MEP amplitude corresponds to increased BF MEP amplitude and decreased SIT. In this subject, 120% RMT equated to 72, 66 and 78% maximal stimulator output in VL, RF and VM, respectively. This indicates that in some subjects, at 120% RMT, coactivation becomes apparent. Coactivation is problematic in the study of fatigue since quantification of cortical VA is essential. At higher stimulus intensities, such as those derived in the relaxed muscle from RMT, SIT during voluntary contraction may be underestimated because of increased contribution of antagonistic muscles [31] without corresponding augmentation of *quadriceps femoris* MEP amplitude.

#### ***Active motor threshold***

Selected stimulus intensity at 120% AMT is significantly lower than stimulus–response curves at 10 and 20% MVC. All lower-limb studies employing AMT as a basis for TMS intensity determination utilized intensities much lower than the IFCN comparison equivalent of 170% AMT [5], recommendations much closer to a 10% MVC stimulus–response curve in this study (Table 1). As with RMT, the use of 6, 8 or 10 stimuli at each stimulus intensity when determining AMT did not affect the stimulus intensity selected.

Background EMG activity varies between individuals and also between muscles at a given contraction intensity; in some cases normal peak-to-peak amplitudes vary by >500% between subjects in the same muscle. Thus, the appropriateness of the common use of a fixed MEP amplitude to determine the presence of a MEP in evaluating AMT at different contraction intensities and in different subjects and/or muscles must be investigated. Boltzmann modeling indicates high inter-subject variability in evolution from no evoked MEP response to a maximal one (i.e. *k*; see Table 3). Some subjects demonstrated what could be characterized as a threshold from which no response immediately became a maximal one while in other subjects MEP amplitude gradually increased to maximum as stimulus intensity increased. Comparison with stimulus–response curves indicates that using AMT to determine stimulus intensity may result in submaximal MEP responses that are situated on the rising part of the Boltzmann curve. Unlike the use of maximal muscular responses to neural stimulation allowing serial or between-subject comparisons, comparison of submaximal evoked responses may introduce additional confounding factors. It remains to be established whether submaximal and maximal MEP responses and their evolution (e.g. with fatigue) are similar, particularly since preliminary indications from upper- [1] and lower-limb [3] studies suggest this may not always be the case. The evaluation of cortical VA may also be affected by the use of stimulus intensities derived from AMT (e.g. 120%). Stimulus intensity at 120% AMT was non-significantly lower than that determined from a 50% MVC stimulus–response curve and this might result in delivery of TMS at a submaximal intensity during contractions between 50 and 100% MVC and result in underestimated SIT. The effect on estimated resting twitch, calculated from the linear regression of three SITs from three different contraction intensities in this range and acceptable if r > 0.9 [48,49], and subsequent estimation of cortical VA are unknown.

Generally, AMT is evaluated in voluntary contractions at 5 or 10% MVC in the upper limbs [50-53]. In lower limbs, Kalmar and Cafarelli [17] and Hilty et al. [16] used 3% MVC and found higher AMT than in Weier et al. [12] and the present study, the latter two having employed contractions at 10% MVC. This is consistent with Boltzmann modeling showing decreased stimulus intensity to evoke a MEP of half maximal amplitude (i.e. *S*50; see Table 3) as contraction intensity increases.

#### ***Stimulus–response curves***

All stimulus–response curves demonstrated a Boltzmann sigmoidal relationship, thus permitting the use of a stimulus–response curve to identify maximal MEP amplitudes and directly determine optimal diagnostic TMS stimulus intensity [5]. Modeling of data indicated that estimated maximal MEP amplitude was lower at 10% MVC than at other contraction intensities although this was only significant in RF. Stimulus intensity to evoke a MEP of half maximal amplitude also decreased as contraction intensity increased. Determining stimulus intensity during contractions at 50% MVC would appear to be appropriate since evoked MEP responses at this contraction intensity are theoretically maximal [20,25,31] and both this and higher contraction intensities are used to determine cortical VA. A concern, however, is that an extended series of such contractions may produce measurable effects of fatigue, and consequently, that residual effects of fatigue may be present during a subsequent protocol as reported in a recent study [6]. The lack of difference between stimulus intensity as determined by stimulus–response curves at 20 and 50% MVC and the similar maximal MEP amplitudes as determined by Boltzmann modeling suggest that in the *quadriceps femoris*, a stimulus–response curve at 20% MVC is appropriate to determine TMS intensity when the aim is to evaluate fatigue-related parameters such as VA.

### Comparison of muscles

Studies determining stimulus intensity during contractions have often used normalized MEP amplitude or area of a given size as criteria [7,8,19,20]. For example, Sidhu et al. [20] selected an intensity that produced the largest RF MEP with the stipulations that this must be at least 50% Mmax and that antagonist BF MEP amplitude be less than 10% raw RF MEP amplitude. In VL and VM in the present study, only 2 of 8 and 3 of 8 subjects, respectively, satisfied the requirement that MEP amplitude be ≥50% Mmax. In the case where several quadriceps muscles are examined, the latter criterion is ambiguous. BF may often be greater than 10% raw MEP amplitude in at least one knee extensor muscle and for one subject in the present study this was the case in all muscles at almost all stimulus intensities evaluated and also at almost all coil positions evaluated in the determination of optimal coil position.

There was no difference in selected stimulus intensity between muscles as determined by any method. This suggests that one muscle could be used as a surrogate for other quadriceps muscles. RF alone has frequently been used to determine quadriceps stimulus intensity [6,8,19,20]. When RF is normalized, MEP amplitude is larger than for either VL or VM due to consistently smaller Mmax in the RF and little differences in raw MEP amplitude. The presentation of normalized RF MEP amplitudes instead of VL and VM may give the impression of eliciting greater corticospinal drive to the quadriceps muscles. In the present study, this was not due to a greater RF contribution since RMS · Mmax^-1^ was only greater than that of VL and VM at 50% MVC and normalized RF MEPs are larger than VL and VM at all contraction intensities. RF may not be an ideal surrogate because of the difficulty in recording clear M waves in this muscle. Furthermore, RF is the sole biarticular muscle of the *quadriceps femoris*, and thus, may not be representative of the muscle group.

An important limitation to the protocol is that it was not conducted on a second day to investigate the day-to-day variability of the methods employed in this study. Further investigations are required to establish whether the different methods employed to evaluate TMS parameters with fatigue are reproducible on different days. The present study also used a maximal response in contracting muscle as a reference point to evaluate multiple fatigue-related TMS parameters since this provides important insights into the manifestation and development of fatigue; however, recent studies suggest that TMS responses elicited by a submaximal stimulus intensity may also further understanding of fatigue mechanisms [1-3]. This reinforces the necessity of selecting an appropriate method to determine TMS intensity directly related to the parameters being investigated. In the context of the evaluation of cortical voluntary activation and corticospinal excitability with fatigue, maximal responses as investigated in this study are pertinent. In other research and diagnostic areas employing TMS, this may not be the case, and methods such as RMT and AMT may be the methods of choice for determining optimal stimulus intensity. Further studies must also determine the specific relevance of TMS-induced maximal and submaximal responses in both healthy and clinical populations in the context of fatigue, including the manner in which this affects measures of cortical voluntary activation and both excitatory and inhibitory mechanisms.

## Conclusions

Percentages of AMT and RMT have often been employed to determine TMS intensity in studies evaluating fatigue; however, these methods do not accurately identify the minimum stimulus intensity to elicit MEPs of maximal amplitude in the *quadriceps femoris*. Thus, they may be inappropriate for cortical excitability and voluntary activation assessment. The potential for increased coactivation and discomfort at 120 and 130% RMT and possible underestimation of evoked responses at 120% AMT preclude their use. There are minor differences between selected stimulus intensity (lower at 50% MVC for VL only) from stimulus–response curves at 20 and 50% MVC. Both MEP amplitude at selected stimulus intensity and estimated maximal MEP amplitude determined from these stimulus–response curves are similar. This indicates that a stimulus–response curve performed at 20% MVC is a suitable method of determining TMS stimulus intensity while reducing the risk of inducing fatigue compared to methods at a higher percentage of MVC. From the present study, it is also concluded that determining stimulus intensity from a single muscle is acceptable in the *quadriceps femoris*.

## Abbreviations

AMT: Active motor threshold; ANOVA: Analysis of variance; BF: *Biceps femoris*; EMG: Electromyography; IFCN: International Federation of Clinical Neurophysiology; k: Slope parameter (Boltzmann modeling); Mmax: Maximal M-wave amplitude; MEP: Motor-evoked potential; MEPmax: Estimated maximal MEP amplitude (Boltzmann modeling); MVC: Maximal voluntary force; RMT: Resting motor threshold; RF: *Rectus femoris*; S: Stimulus intensity (Boltzmann modeling); S50: Stimulus intensity to produce a response half that of MEPmax (Boltzmann modeling); SIT: Superimposed twitch; TMS: Transcranial magnetic stimulation; VL: *Vastus lateralis*; VM: *Vastus medialis.*

## Competing interests

The authors declare that they have no competing interests.

## Authors’ contributions

JT, MG, TR, SV and GYM all contributed to the project conception, data collection and analysis and manuscript preparation. All authors read and approved the final manuscript.
